# Impact of endogenous and exogenous nitrogen species on macrophage extracellular trap (MET) formation by bone marrow–derived macrophages

**DOI:** 10.1007/s00441-023-03832-z

**Published:** 2023-10-04

**Authors:** Dominika Drab, Michal Santocki, Malgorzata Opydo, Elzbieta Kolaczkowska

**Affiliations:** 1 Laboratory of Experimental Hematology, Institute of Zoology and Biomedical Research, Jagiellonian University, 30-387 Krakow, Poland; 2 Doctoral School of Exact and Natural Sciences, Jagiellonian University, Krakow, Poland

**Keywords:** Bone marrow-derived macrophages, Innate immunity, Macrophage extracellular traps, Nitric oxide, Lipopolysaccharide

## Abstract

Macrophage extracellular traps (METs) represent a novel defense mechanism in the antimicrobial arsenal of macrophages. However, mechanisms of MET formation are still poorly understood and this is at least partially due to the lack of reliable and reproducible models. Thus, we aimed at establishing a protocol of MET induction by bone marrow–derived macrophages (BMDMs) obtained from cryopreserved and then thawed bone marrow (BM) mouse cells. We report that BMDMs obtained in this way were morphologically (F4/80^+^) and functionally (expression of inducible nitric oxide (NO) synthase and NO production) differentiated and responded to various stimuli of bacterial (lipopolysaccharide, LPS), fungal (zymosan) and chemical (PMA) origin. Importantly, BMDMs were successfully casting METs composed of extracellular DNA (extDNA) serving as their backbone to which proteins such as H2A.X histones and matrix metalloproteinase 9 (MMP-9) were attached. In rendered 3D structure of METs, extDNA and protein components were embedded in each other. Since studies had shown the involvement of oxygen species in MET release, we aimed at studying if reactive nitrogen species (RNS) such as NO are also involved in MET formation. By application of NOS inhibitor — L-NAME or nitric oxide donor (SNAP), we studied the involvement of endogenous and exogenous RNS in traps release. We demonstrated that L-NAME halted MET formation upon stimulation with LPS while SNAP alone induced it. The latter phenomenon was further enhanced in the presence of LPS. Taken together, our findings demonstrate that BMDMs obtained from cryopreserved BM cells are capable of forming METs in an RNS-dependent manner.

**Supplementary Information:**

The online version contains supplementary material available at 10.1007/s00441-023-03832-z.

## Introduction

Macrophages constitute a heterogenous population of innate immune cells located in different tissues and organs that play vital functions in homeostasis, surveillance, and immune responses (Wynn et al. 2013). Macrophages are well defined for their ability to phagocytose pathogens and produce various cytokines and antimicrobial proteins, enzymes, reactive oxygen species (ROS) and reactive nitrogen species (RNS) that are used to eliminate microbial invaders (Davies et al. 2013). Recently a new weapon was identified in the repertoire of macrophage defense arsenal — the capacity to cast extracellular traps (ETs) (Chow et al. 2010). ET formation was first identified in neutrophils which are able to release neutrophil extracellular traps (NETs) (Brinkmann et al. 2004; Kolaczkowska et al. 2015). Subsequently, granulocytes such as eosinophils (Yousefi et al. 2012), basophils (Yousefi et al. 2015), and mast cells (Von Köckritz-Blickwede et al. 2008) were also shown to release them. However, discovery that monocytes (Haritha et al. 2019) and macrophages (Chow et al. 2010) are also able to form ETs (hence METs) entrapping and killing various microorganisms was a surprise as they represent a separate developmental branch of myeloid cells. Over the years, the composition of ETs and mechanisms involved in their production were mainly investigated in the case of neutrophils (Papayannopoulos 2018) while knowledge on ET formation by macrophages is still fragmentary. The main component of macrophage extracellular traps (METs) is extracellular DNA (extDNA) which constitutes the DNA scaffold to which histones and granular proteins are attached. Thus far, they were identified to include matrix metalloproteinases (MMPs): MMP-1, -7, -8, -9, and -12 (Sharma et al. 2017), lysozyme (Boe et al. 2015), lactoferrin (Doster et al. 2018b), myeloperoxidase (Liu et al. 2014) and neutrophil elastase (NE) (Je et al. 2016). This indicates that METs and NETs share many structural and functional similarities but also explains why studies on METs are challenging and hardly possible *in vivo*. Some studies have indirectly shown that MET formation takes place in pathological conditions *in vivo*. METs have been identified in different diseases such as atherothrombosis and they have been detected in atherosclerotic plaques by colocalization of citrullinated histone H3 (CitH3) with CD68^+^ (human macrophage marker) cells (Pertiwi et al. 2019). MET formation seems to occur also in acute kidney injury in mice, as it was observed in the renal tubules in the areas with F4/80^+^ cells where CitH3 and extDNA signal was also present (Okubo et al. 2018). Tumor infiltrating macrophages can also contribute to the overall ET content as in CD68^+^ cells rich areas the presence of CitH3^+^ was observed in patients with pancreatic neuroendocrine tumors (Xu et al. 2021).

Most studies on MET formation have been performed on macrophage-like cell lines such as J774A.1, RAW 264.7, THP-1 while fewer on primary macrophages or macrophages that originate from monocyte precursors in the bone marrow (Doster et al. 2018a). However, some studies regarding MET formation have also been performed on bone marrow-derived macrophages (BMDMs) (Bonne-Année et al. 2014; Mónaco et al. 2021; Gao et al. 2022) and human monocyte-derived macrophages (HMDMs) (Rayner et al. 2018; Zhang et al. 2019) all proving their ability to release METs. Despite the fact that transformed or immortalized macrophage-like cell lines are commonly used in immunological investigations, they have several limitations (e.g., genetic drift/loss of genes due to subculturing) that affect their biology (Andreu et al. 2017). In addition, their immortality is unphysiological, and furthermore, cell lines can express distinctive gene patterns that are not present in cells in *in vivo* conditions (Levenson et al. 2018). Therefore, macrophages from cell lines can significantly differ from primary macrophages and might be physiologically irrelevant (Tedesco et al. 2018). On the other hand, continuous experiments on freshly isolated primary cells represent a challenging model in a day-to-day laboratory practice. To find a satisfactory compromise, we established a method of differentiating macrophages from their precursors collected from bone marrow, the so-called BMDMs (Marim et al. 2010; Bonne-Année et al. 2014). We tested the cells obtained and differentiated from pre-frozen (cryopreserved), and then thawed out, bone marrow. Additional advantage of working with BMDMs is the fact that they are not polarized with predetermined functions (e.g., pro- or anti-inflammatory) and they also divide when in culture. The latter is due to the fact that they originate from monocyte precursors residing in the bone marrow which renew different macrophage populations during organism lifetime (Ginhoux and Jung 2014). Furthermore, BMDMs are naïve as they have not been exposed to any antigens and their isolation from the bone marrow is performed in a sterile manner (Marim et al. 2010). Importantly, bone marrow cells can be cryopreserved and stocked in liquid nitrogen and can be readily used for macrophage differentiation when needed (Marim et al. 2010).

Although various microbial organisms (bacteria, parasites and fungi) and chemical inducers, including phorbol myristate acetate (PMA), are capable of triggering MET release (Doster et al. 2018a), there is a lack of consistent knowledge about mechanisms involved in their formation. Currently, attempts are being made to investigate the same mechanisms that have been previously studied and described in the case of NETs. In line with this, involvement of histone hypercitrullination allowing for chromatin decondensation facilitating NET release (Lewis et al. 2015) was also described in METs. Regarding NETs, protein arginine deiminase 4 (PAD4) enzyme catalyzes the conversion of positively charged arginine residues into neutral citrulline residues in histones loosening the chromatin structure. In MET formation, involvement of another isoform of the same enzyme - PAD2 was reported upon stimulation of RAW 264.7 macrophages with TNF-α (Mohanan et al. 2013). Interestingly, some MET studies reported that under different stimuli, MET formation was independent of PAD activity. For example, Rayner et al. 2018 demonstrated that M1-polarized HMDMs released METs in a PAD-independent manner upon stimulation with hypochlorous acid (HOCl), PMA, IL-8 or TNFα, and even pretreatment with a pan-PAD inhibitor Cl-amidine did not decrease MET release. Among other identified mechanisms, activation of NADPH oxidase and subsequent ROS generation were also confirmed to be required for MET release as they are for NETs (Aulik et al. 2012; Doster et al. 2018b). However, the involvement of reactive nitrogen species confirmed thus far to participate in NET release (Manda-Handzlik et al. 2020) was not yet studied during MET formation. RNS are a family of antimicrobial molecules derived primarily from nitric oxide (NO) produced via the enzymatic activity of inducible nitric oxide synthase 2 (iNOS or NOS2) (McNeill et al. 2015). The enzyme is expressed in stimulated, but not resting, macrophages that have been exposed for example to lipopolysaccharide (LPS), a component of the outer membrane of Gram-negative bacteria and one of the strongest immune-stimuli (Leiva-Salcedo et al. 2011).

Therefore, the aim of the current study was to evaluate the capacity of cryopreserved BMDMs to cast METs upon exposure to various types of immune stimulants (yeast, bacterial or chemical) and then evaluate the role of NO in MET production. Herein, we report that BMDMs are able to form METs after stimulation with LPS as well as zymosan or PMA, and that both endogenous as well as exogenous nitric oxide is involved in this process or can induce it, respectively.

## Materials and methods

### Bone marrow isolation

Bone marrow was obtained from femurs and tibias of 14–18-week-old male mice of the C57BL/6J strain (Charles River, Germany). Mice were anesthetized by intraperitoneal injection of anesthetics, a mixture of ketamine hydrochloride (200 mg/kg b.w.; Biowet Pulawy, Poland) and xylazine hydrochloride (10 mg/kg b.w.; aniMedica, Germany). All experimental animal protocols were approved by the Local Ethical Committee No. II in Kraków (294/2017) and were in compliance with the EU Animal Care Guidelines. Mice were then sacrificed by cervical dislocation. After euthanasia, the fur was sprayed with 70% ethanol for disinfection and mice were dissected. Femurs and tibias were isolated with scissors, cutting the tibias below the knee joints and the femurs near the hip joints. Muscles attached to the bones were removed with scissors and Kimwipes (Kimberly Clark KIMTECH Science, USA), then the bones were placed in a 15 ml polystyrene tube (NEST Scientific, USA) containing sterile Hank’s Buffered Salt Solution without calcium and magnesium ions, HBSS (-) (Lonza Bioscience, USA) and kept on ice. In the next step, under sterile conditions, the bones were placed in sterile Petri dish (60/15 mm) containing 70% ethanol for 1 min, and then rinsed with sterile HBSS (-). Then both epiphyses of the bones were cut with scissors and bones were placed in a new Petri dish containing cold RPMI 1640 (++) (Lonza Bioscience, USA) with 10% fetal bovine serum (FBS) (Biowest, USA), 2 mM L-glutamine, 2% (v/v) antibiotics, penicillin and streptomycin (Sigma-Aldrich, USA) (++ corresponds to RPMI 1640 with 10% FBS, 2 mM L-glutamine and 2% (v/v) antibiotics). The bones were flushed using a syringe (Polfa Lublin, Poland) with 25G injection needle (Terumo Agani, China) filled with cold RPMI 1640 (++) and the bone marrow was flushed out into a Petri dish and then dissociated using a syringe with a 20G needle (Terumo Agani, China). For this purpose, the bone marrow was collected and dissociated several times with a syringe until a homogeneous suspension was obtained. The uniform bone marrow suspension was transferred to a 15 ml polystyrene tube and filled up with RPMI 1640 medium (++) to the maximum volume and centrifuged for 6 min at 275 × g at 4 °C. After centrifugation, the supernatant was discarded and the erythrocyte lysis was performed, for this purpose 1 ml of 0.2% NaCl solution was added, pipetted and then 4 ml of 0.2% NaCl solution was added. In the next step, 5 ml of 1.6% NaCl solution was added and the suspension was centrifuged for 7 min at 319 × g at 4 °C. Then, the cells were resuspended in 1 ml of RPMI 1640 (++) and counted.

### Preparation of bone marrow cells for experiments

Cryopreservation and thawing of bone marrow cells; fresh bone marrow cells were counted and resuspended in freezing medium containing 90% FBS and 10% RPMI 1640 (++) to obtain 6 × 10^6^ cells/ml, and each milliliter of the suspension was transferred to an individual cryovial (NEST Scientific, USA) and additionally 100 µl of DMSO (Sigma-Aldrich, USA) was added. Such bone marrow cells were cryopreserved in a two-step freezing procedure, in which the bone marrow cells were first maintained in −80˚C for 24 h and then transferred to liquid nitrogen. At the time of experiment, the bone marrow cells were thawed at 37˚C in water bath until the suspension was entirely thawed. BM cell viability was verified by trypan blue (Sigma-Aldrich, USA) exclusion test and it was 85 ± 2% for the cryopreserved cells *versus* 90 ± 3% for the freshly differentiated cells. Next, cell suspensions were transferred to 15 ml polystyrene tubes containing warm RPMI (++), centrifuged at 200 × g for 5 min and then the cell suspension was resuspended in BMDM differentiation medium (Marim et al. 2010) composed of RPMI 1640 medium supplemented with 20% FBS (Biowest, USA), 100 U/ml penicillin, 100 µg/ml streptomycin, 2 mM L-glutamine (Sigma-Aldrich, USA) and 30% LCCM (preparation of LCCM is described below).

### L-929 cell conditioned medium (LCCM) preparation

L-929 murine fibroblast cell line (American Type Culture Collection, USA) was used to collect L-929 cell conditioned medium which serves as a source of macrophage colony-stimulating factor (M-CSF) (Englen and Lehnert 1995). Fibroblasts were maintained in RPMI 1640 medium, supplemented with 10% FBS and 2 mM L-glutamine with 2% (v/v) antibiotics, penicillin and streptomycin. Fibroblasts were grown in T-25 flasks (Greiner Bio-One, Germany) in a humidified incubator with 5% CO_2_ at 37 °C (Thermo Fisher Scientific, USA). Fibroblasts were passaged at 90% confluence with a cell scraper (Biologix, USA). From the 5^th^ passage, the cells were maintained in T-75 flasks (Greiner Bio-One, Germany) for the LCCM production and left without changing the medium for the next 10 days. After this time, L-929 supernatants were aspirated with a serological pipette and transferred to 15 ml polystyrene tubes. The supernatants were then centrifuged for 10 min at 366 × g at 20 °C. After centrifugation, the supernatants were transferred to 50 ml polystyrene tubes (NEST Scientific, USA) and filtered using a 0.22 µm filter (NEST Scientific, USA). Aliquoted LCCM was stored at −20 °C.

### BMDM differentiation, culture and proceeding

Bone marrow-derived macrophages were used in all experiments and cryopreserved thawed bone marrow cells were used for BMDM generation. A protocol of Marim et al. (2010) was followed with some modifications as detailed below. Bone marrow cells were seeded and cultured in T-25 flasks (Greiner Bio-One, Germany) with added BMDM differentiation medium (collected as described above) in a humidified incubator with 5% CO_2_ at 37 °C (Thermo Fisher Scientific, USA). Four days after seeding the cells, 5 ml of fresh BMDM differentiation medium was added to the flasks and left in the incubator for another 3 days. The process of macrophage differentiation took 7 days. After differentiation, the BMDM differentiation medium was discarded, and BMDMs were washed with PBS (Sigma-Aldrich, USA) and detached with cell scraper upon resuspension in 5 ml of RPMI 1640 (++); then they were centrifuged at 200 × g for 5 min. Next, BMDMs were seeded and stimulated in R10/5 medium, i.e. RPMI 1640 medium supplemented with 10% FBS, 2 mM L-glutamine, 100 U/ml penicillin, 100 µg/ml streptomycin and 5% LCCM (R10/5: 10 refers to the percentage of FBS and 5 to the LCCM content).

### Confirmation of BMDMs differentiation by flow cytometry

After 7 days of differentiation, BMDMs were detached from flasks with a cell scraper and centrifuged for 5 min at 200 × g at 20 °C. The supernatants were discarded and cell pellets were resuspended in HBSS (+) with calcium and magnesium ions (Lonza Bioscience, USA). Cells were counted and brought to a density of 5 × 10^5^ − 1 × 10^6^/ml cells per Eppendorf tube. Supernatants were removed and 100 µl of Fc block (BD Biosciences, USA) was added to block non-specific binding of antibodies to receptors for Fc fragments on the macrophage surface. Eppendorf tubes were left on ice for 15 min and then centrifuged at 2325 × g for 5 min and at 4 °C (in all subsequent steps the same centrifugation was followed). The supernatants were then removed and cells were stained with rat anti-mouse F4/80 antibodies conjugated with PE (1:100, eBioscience, USA), Alexa Fluor 488 rat anti-mouse F4/80 antibodies (1:100, eBioscience, USA), PE rat anti-mouse CD11a/CD18 (LFA-1; leukocyte function associated antigen-1) antibodies (1:100, BioLegend, USA). PE-conjugated rat IgG2a kappa PE antibodies were used as the isotype control (1:100, BioLegend, USA). Incubation with antibodies lasted 15 min on ice in the dark. After incubation, cells were centrifuged and washed once by resuspending the cells in 500 µl of staining buffer prior to centrifugation. The staining buffer consisted of HBSS (+) supplemented with 3% FBS. The pellets were resuspended in staining buffer (500 µl of staining buffer for controls and 300 µl for the experimental samples) and transferred to FACS tubes (BD Biosciences, USA). Data were acquired with a FACSCalibur (Becton Dickinson, USA) and analyzed with WinMDI 2.9 Software.

### Confirmation of BMDMs differentiation by immunocytochemistry

To confirm differentiation of BMDMs, we also verified expression/presence of F4/80 marker on the surface of differentiated macrophages with immunocytochemistry. Cells were fixed with 4% paraformaldehyde (PFA) (AlfaAesar, Germany) and blocking of non-specific antibody binding sites was performed with 3% of bovine serum albumin (BSA) in PBS for 45 min at room temperature in a humid chamber. Staining with PE rat anti-mouse F4/80 antibody (eBioscience, USA) diluted 1:100 in 1% BSA was performed overnight at 4˚C in a humid chamber. Sytox Green (Molecular Probes, Inc., USA) at a concentration of 5 µM was used to stain extracellular DNA (extDNA). Vectashield mounting medium (Vector Laboratories, USA) was used to mount the cells.

### Stimulation of BMDMs

To induce METs, the following stimulants were used: lipopolysaccharide (LPS) from *Escherichia coli* serotype O111:B4 (Sigma-Aldrich, USA), Zymosan A from *Saccharomyces cerevisiae* (Sigma-Aldrich, USA), phorbol 12-myristate 13-acetate (PMA) (Sigma-Aldrich, USA). The stimulants were added to BMDMs at time 0. In some experiments, the cells were pretreated with N(gamma)-nitro-L-arginine methyl ester hydrochloride — L-NAME (Abcam, Cambridge, UK) or NO donor S-Nitroso-N-acetyl-DL-penicillamine — SNAP (Sigma-Aldrich, USA) 30 min prior to stimulation with LPS or zymosan. Subsequent analyses were performed 18 h after stimulation with LPS, zymosan or PMA. BMDMs were seeded in 96-well plates and 24- or 96-well plates (NEST Scientific, USA) on coverslips (Thermo Fisher Scientific, USA) for further immunocytochemical analysis, and then left to adhere for at least 12 h before any further experimental procedure. BMDMs were stimulated with LPS (1 μg/ml), zymosan (50 μg/ml) or PMA (156 ng/ml) (Chow et al. 2010) and left for overnight incubation.

### Visualization of extracellular DNA of METs

The ability of differentiated live unfixed macrophages (25 × 10^3^/well) to form METs was estimated by live cell fluorescence imaging with Sytox Green. Briefly, 10 μl of Sytox Green stain (5 µM) in PBS was added to each well of a 96-well plate immediately after overnight incubation with stimulants. Images (at least three from each experimental group) were taken from under an inverted fluorescence microscope (Zeiss Axio Vert. A1 FL, Germany). The intensity of Sytox Green staining was analyzed and measured with adjusted contrast to exclude background autofluorescence signal, and a minimum brightness threshold was set to yield only positive staining and applied to all images. Thresholded images were converted to binary (black and white) and nuclei of Sytox-positive cells alone were removed in the ImageJ software, and the area per field of view covered by positive fluorescent staining (black) from MET forming cells and METs, was calculated with ImageJ software (Supplementary Fig. 2).

### Visualization of MET proteins and extDNA by immunocytochemistry

After BMDM stimulation as described above, cells/METs seeded on coverslips were fixed in 4% PFA at room temperature and washed with PBS. In order to detect the specific components of METs, goat anti-MMP-9 polyclonal antibodies conjugated with Alexa Fluor 647 (IgG, Santa Cruz Biotechnology, CA, USA) and mouse anti-H2A.X monoclonal antibodies conjugated with Alexa Fluor 568 (IgG1 kappa light chain, Santa Cruz Biotechnology, CA, USA) were used. Specimens were blocked with 3% BSA to prevent unspecific antibody binding and stained with anti-MMP-9 and anti-H2A.X antibodies diluted in 1% BSA in a ratio of 1:50 and 1:100, respectively. Staining with antibodies was performed overnight at 4˚C. After overnight incubation, the coverslips were washed with PBS and Sytox Green stain (5 µM) was added for 5 min to stain extracellular DNA. Next, the coverslips were mounted on slides with Vectashield mounting medium. Images were taken from a confocal microscope as described in detail below.

### Visualization and 3D imaging of METs

METs were visualized with ZEISS Axio Examiner.Z1 upright microscope equipped with a metal halide light source (AMH-200-F6S; Andor, Oxford Instruments) and with DSD2 spinning-disk confocal module (Andor, Oxford Instruments). Images were taken in RFP (red fluorescent protein), GFP (green fluorescent protein), Cy5 (cyanine-5) and DAPI channels for histone H2A.X, extracellular DNA, MMP-9 and DNA, respectively. The components were stained with the antibodies as described in the section “Visualization of MET proteins and extDNA by immunocytochemistry”. In order to spatially visualize the structure of METs, a series of images were taken from the slide in the z-stack mode (z-stack thickness 100 μm). In the final stage, each obtained 3D MET image was analyzed and edited in the Imaris software (Imaris Software, Oxford Instruments). Z-stacks were imported into Imaris and 3D rendering of MET morphology was done using surfaces functions with default colors appropriate to each fluorochrome used to visualize histone H2A.X (red), MMP-9 (purple) and extDNA (green). The 3D reconstruction was converted into the 3D video (Supplementary Video 1).

### NO assay

In order to determine the ability of BMDMs to produce NO, the concentration of its final products — nitrates and nitrites, was determined using the Griess colorimetric assay. In accordance with the protocol (Kolaczkowska et al. 2010), 100 μl of supernatant from each well/experimental group was added in triplicate to a new 96-well plate, and then 50 μl of 1% sulfanilamide (Sigma Aldrich, USA) in 5% phosphoric acid (POCH, Poland) — Griess A reagent — and 50 μl of 0.1% N-(1-Naphthyl)ethylenediamine (Sigma Aldrich, USA) in distilled water — Griess B reagent was added. Absorbance was measured at a wavelength of 570 nm in a spectrophotometric microplate reader (Tecan, Männedorf, Switzerland). The NO concentration was calculated from the standard NO curve, which was prepared by serial dilutions of 2 mM sodium nitrite solution (POCH, Poland) in PBS.

### Intracellular staining of iNOS

After stimulation, cells seeded on coverslips were fixed and permeabilized by washing in a Triton containing PBS solution for 5 min. After blocking, the cells were labeled with rabbit monoclonal anti-iNOS antibodies (diluted 1:500; IgG, Abcam, UK) and incubated overnight at 4^◦^C. The slides were then washed in PBS and incubated with the secondary goat anti-rabbit IgG (H+L) (Cy3) antibody diluted 1:300 (Jackson Immuno Research Laboratories, Inc., USA) for 1 h at room temperature. Then, the coverslips were washed in PBS, stained with Hoechst 33342 (Thermo Fisher Scientific, USA) diluted 1:1000 for 5 min and mounted with a mounting medium prior to confocal microscopy imaging.

### Statistics

The obtained results were analyzed with Student’s *t*-test and one-way analysis of variance (ANOVA) with Tukey post hoc test (GraphPad Prism 6, GraphPad Software, USA). Statistically significant differences were considered at *P ≤ 0.05, **P ≤ 0.01, ***P ≤ 0.001, ****P ≤ 0.0001. Data are presented as mean value ± SD. Different letters indicate statistically significant differences between groups.

## Results

### Confirmation of successful differentiation of bone marrow-derived macrophages (BMDMs) obtained from cryopreserved bone marrow cells

**Fig. 1:**
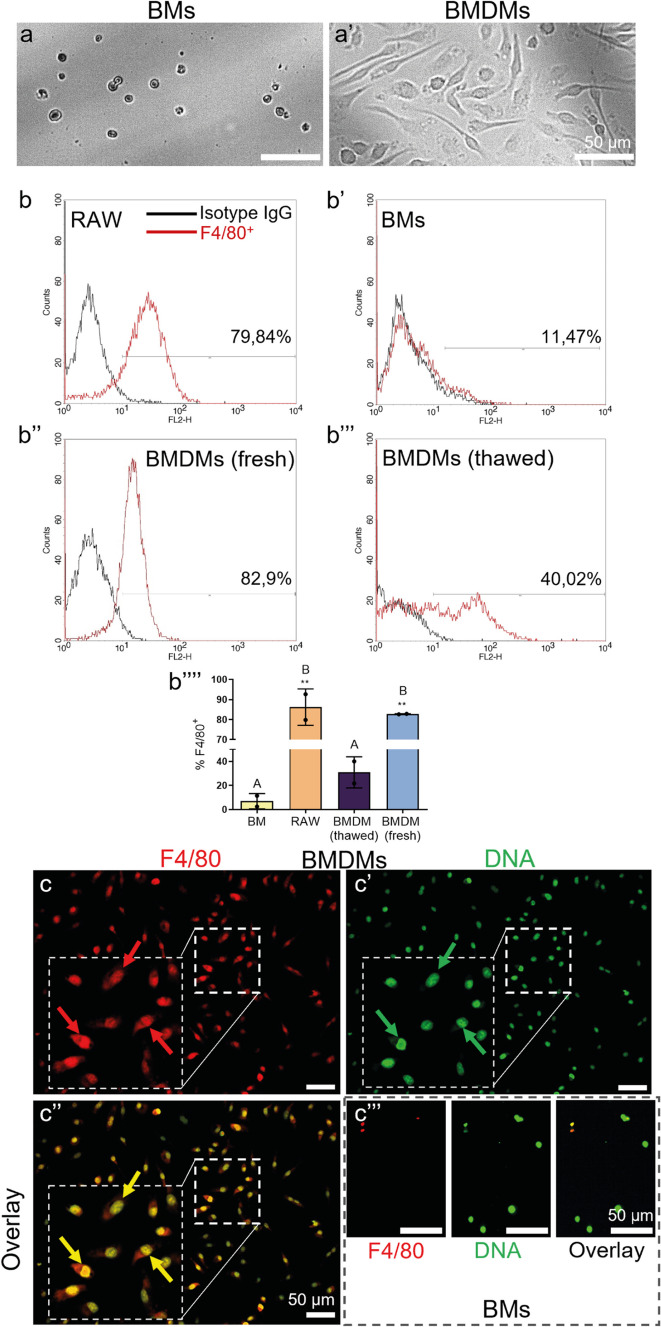
Confirmation of the successful differentiation of bone marrow-derived macrophages (BMDMs) obtained from cryopreserved bone marrow (BM) cells. BMDMs were differentiated from BM cells upon incubation in differentiation medium for 7 days. The BMDMs were then analyzed to confirm their differentiation. **a**–**a’** Morphology of undifferentiated BM cells and BMDMs. Scale bar = 50 μm. **b**–**b’’’** Representative histograms from flow cytometry analysis of macrophage surface marker F4/80 expression **b** on mature RAW 264.7 macrophages serving as a positive control, **b’** undifferentiated BM cells serving as a negative control, **b’’** differentiated fresh BMDMs and **b’’’** differentiated thawed BMDMs. The black line represents the signal of the isotype control antibody and the red line marks the fluorescence intensity of the F4/80^+^ cells. Both antibodies were conjugated with PE and read in channel 2 (FL2-H). Data are shown from a representative experiment. **b’’’’** Quantification of data from **b**–**b’’’** experiments. Asterisks indicate significant differences using unpaired two-tailed Student’s *t* test (**P ≤ 0.01) between various cell types (RAW, fresh BMDMs, thawed BMDMs) versus BMs. Different letters indicate statistically significant differences between groups using a one-way analysis of variance (ANOVA) (post hoc Tukey test). Data are presented as the mean ± SD of duplicate experiments. **c**–**c’’’** Representative immunocytochemical images confirming F4/80 (red) expression on fixed BMDMs. It is contrasted to signal intensity in BM cells. DNA was co-stained with Sytox Green (green) to visualize cell location. Red arrows mark exemplary BMDMs with F4/80 expression, green arrows nuclei of these cells, and yellow arrows mark the double expression/presence of F4/80 and DNA. N = 3. Scale bar = 50 μm

First, we verified if cryopreserved, thawed BM cells can be used for BMDM differentiation. After 7 days of differentiation culture, we observed an alteration in the morphology of bone marrow (BM) derived cells. They were numerous, thus proliferated, were elongated and spread, displaying a typical macrophage morphology (Fig. 1a’). This was in contrast to undifferentiated BM cells, which were smaller and with a round-like morphology (Fig. 1a). The morphology of both cryopreserved and then thawed macrophages, and those differentiated from fresh bone marrow, was the same (not shown). To confirm macrophage differentiation, the expression of the surface marker F4/80 characteristic to mature macrophages was examined by flow cytometry. RAW 264.7 macrophages served as a positive control and almost 80% (79.84%) of them were F4/80^+^ (Fig. 1b). Undifferentiated bone marrow cells weakly expressed that antigen (11.47%; Fig. 1b’) whereas among thawed BMDMs, the percentage of differentiated F4/80^+^ cells was 40.02% (Fig. 1b’’’). However, the latter difference did not reach statistical significance (Fig. 1b’’’’). The expression of F4/80 on differentiated BMDMs was higher when fresh cells were used — 82.9 vs. 40% (Fig. 1b’’). Nevertheless, when we analyzed CD11a/CD18 and F4/80 markers, we observed that over 88% of BMDMs expressed CD11a/CD18 (LFA-1) and over 99% of BMDMs co-expressed it with F4/80 confirming differentiation of the cells (Supplementary Fig. 1). To further clarify the expression of F4/80, its immunocytochemical detection was performed. Clearly, the majority, if not all, of BMDMs expressed the marker as shown in representative images, unlike undifferentiated BMs (Fig. 1c–c’’’).

### LPS, zymosan and PMA trigger MET release

**Fig. 2:**
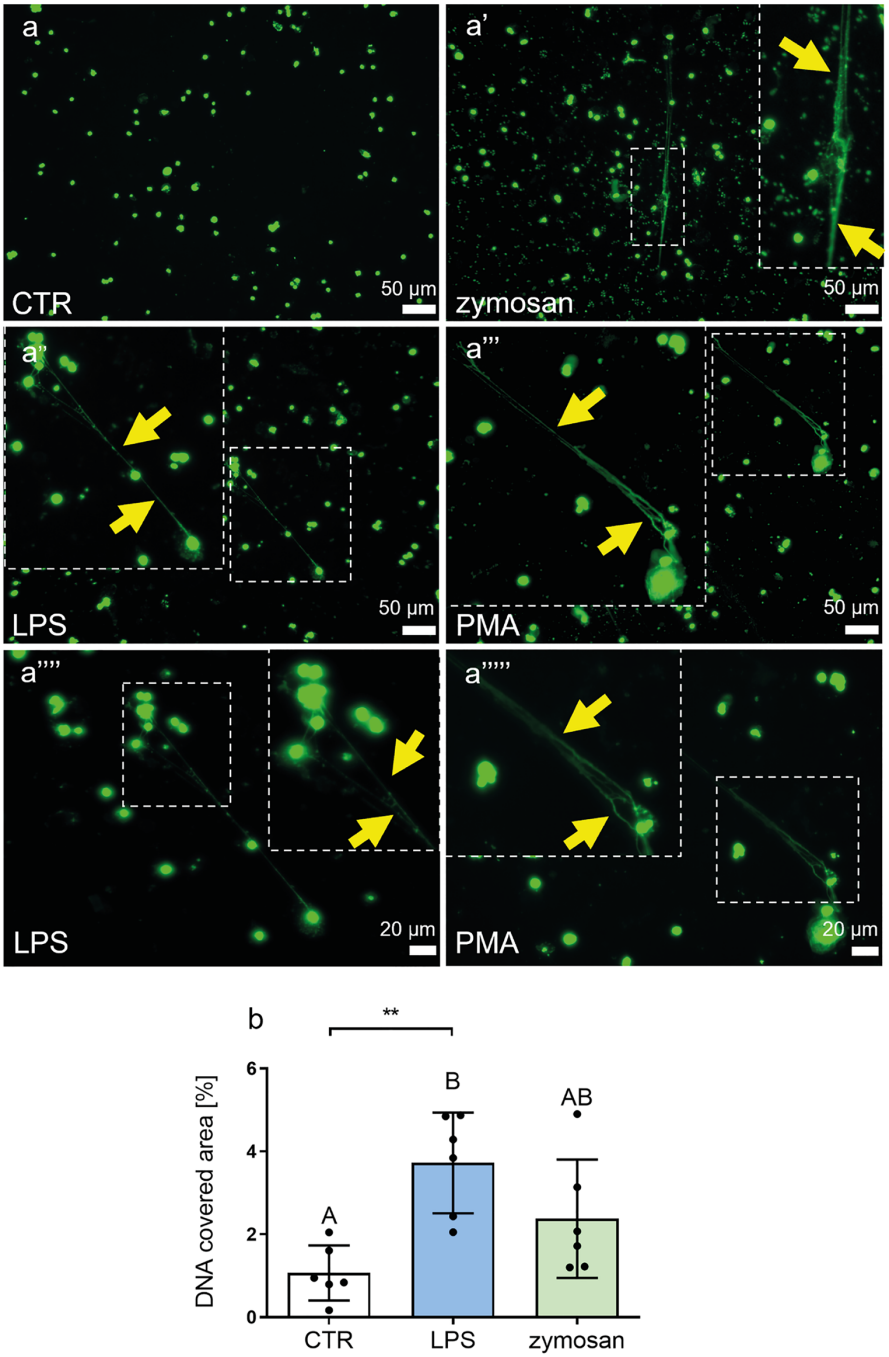
Macrophage extracellular traps (METs) released by bone marrow-derived macrophages (BMDMs) following zymosan, lipopolysaccharide (LPS), and phorbol myristate acetate (PMA) stimulation. **a**–**a’’’’’** Representative images showing METs formed by live unfixed cultures of BMDMs. Differentiated BMDMs were stimulated with LPS (1 µg/ml), zymosan (50 µg/ml) and PMA (156 ng/ml) or left alone (CTR), and incubated overnight to induce METs. METs were visualized by addition of Sytox Green staining DNA. **a’’’’**–**a’’’’’** Images presented in the lower bottom panel were taken with a 40 × objective (scale bar = 20 μm) whereas all other images were taken with a 20 × objective (scale bar = 50 μm). Data are shown from representative experiments. **b** The percentage of DNA covered area as in the images presented in **a**–**a’’’’’** was quantified with ImageJ. Asterisks indicate significant differences using unpaired two-tailed Student’s *t* test (**P ≤ 0.01) between CTR and LPS-stimulated group. Different letters indicate statistically significant differences between groups using a one-way analysis of variance (ANOVA) (post hoc Tukey test). N = 3. Data are presented as the mean ± SD

Subsequently, we investigated if various immunostimulants could induce MET formation by BMDMs. We tested stimulation with bacterial LPS, fungal/yeast zymosan, and chemical PMA as all of them are known to induce NETs and represent diverse spectrum of stimulants. At first, we studied MET release prior to their fixation; METs were stained by addition of Sytox Green which binds to extracellular DNA (extDNA) (Fig. 2a–a’’’’’). The advantage of this approach is unaltered (by fixation) morphology of ETs and maximal (quantitatively-wise) detection of extDNA as some of it is lost when performing washings during the fixation process (Homa et al. 2016). METs appeared as fibers of various length that interconnected one or more cells, and some thinner fibers were also linked into thicker strands. Exemplary METs are indicated in images with yellow arrows (Fig. 2a’–a’’’’’). We then quantified the area covered by extDNA and confirmed the release of METs by LPS (Fig. 2b) and PMA (not shown), but it was weaker in the presence of zymosan (Fig. 2b) showing differential sensitivity of BMDMs towards various stimuli. Additionally, in the case of LPS and PMA stimulation, we observed enlarged nuclei indicative of the first step of ET formation (Tatsiy et al. 2021). To confirm that these were indeed METs, we subsequently stained them for the presence of MET proteins attached to extDNA.

### BMDM METs contain MMP-9 and H2A.X

**Fig. 3:**
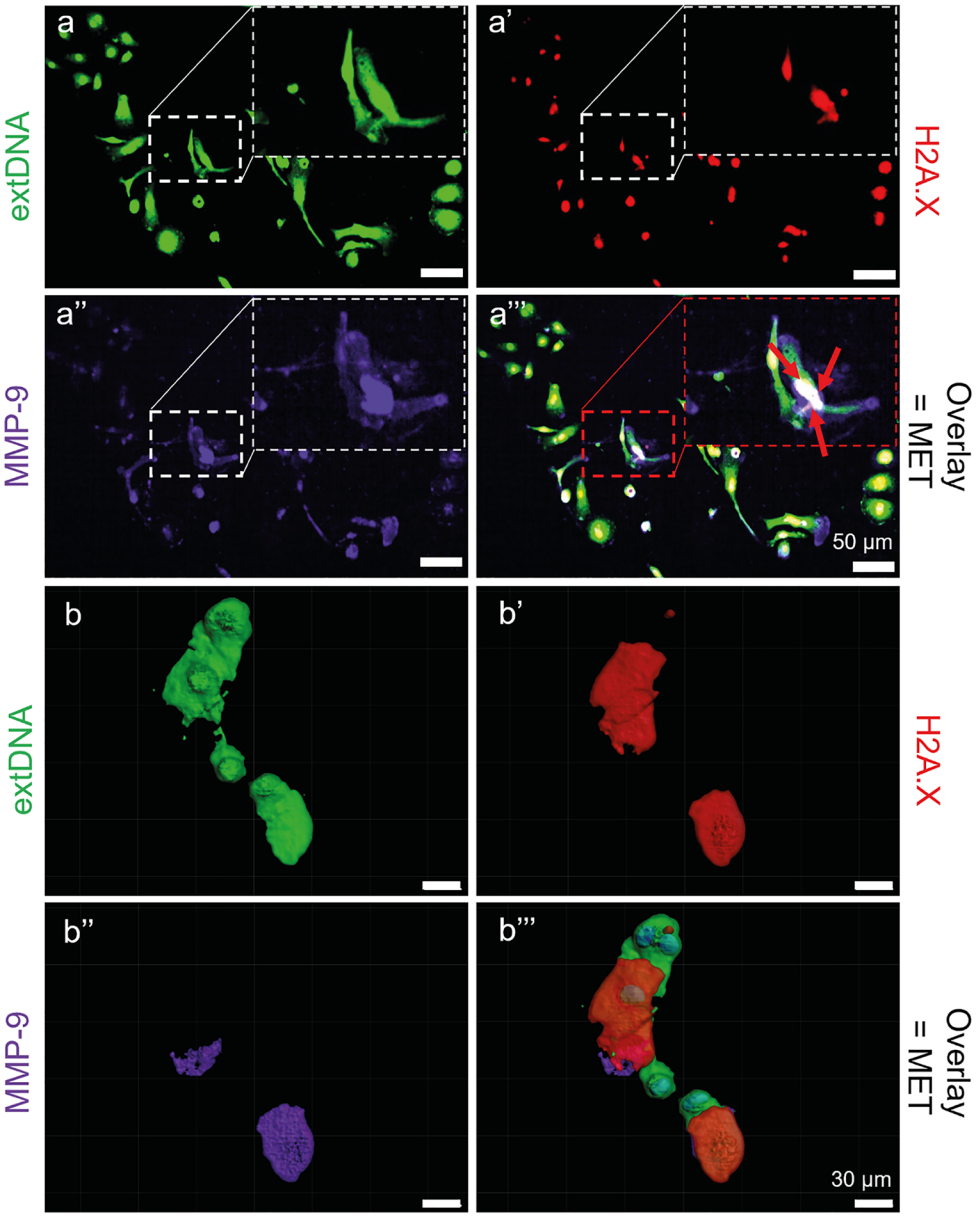
Protein and extracellular DNA complexes are present in the structure of macrophage extracellular traps (METs) formed by bone marrow-derived macrophages (BMDMs) upon stimulation with lipopolysaccharide (LPS). Differentiated BMDMs were stimulated overnight with LPS (1 µg/ml) to induce METs, and subsequently fixed. Then METs were detected immunocytochemically. **a**–**a’’’** Representative images showing the complexity of the MET structure, including the presence of H2A.X histones (red) and MMP-9 (purple) within/along with extracellular DNA (extDNA; green). An exemplary MET is indicated by red arrows (overlay = MET). Scale bar = 50 μm. **b**–**b’’’** Additionally, the three-dimensional (3D) MET structure was reconstructed with Imaris software and METs were visualized in the z-stack mode to create the 3D image of the METs (z-step = 1 µm, z-stack thickness = 100 µm). Scale bar = 30 μm

METs are composed of various nuclear and granular proteins similarly to NETs (Weng et al. 2022). We chose to detect one granular protein (MMP-9) and one nuclear - histone H2A.X, in METs. Immunocytochemistry demonstrated the colocalization of the signal from these two proteins with extDNA (Sytox green) as H2A.X and MMP-9 were detected along the DNA fibers (Fig. 3a–a’’’). In an attempt to reconstruct the 3-dimensional structure of METs, their z-stacks were performed. The reconstructed model confirmed that extDNA constituted the scaffold to which the proteins (H2A.X and MMP-9) were attached, and in fact all three components seem to be embedded in each other as seen in exemplary images (Fig. 3b–b’’’) and video (Supplementary Video 1). The 3D model further confirmed colocalization of the proteins.

### BMDMs express iNOS and produce NO upon LPS and zymosan stimulation

**Fig. 4:**
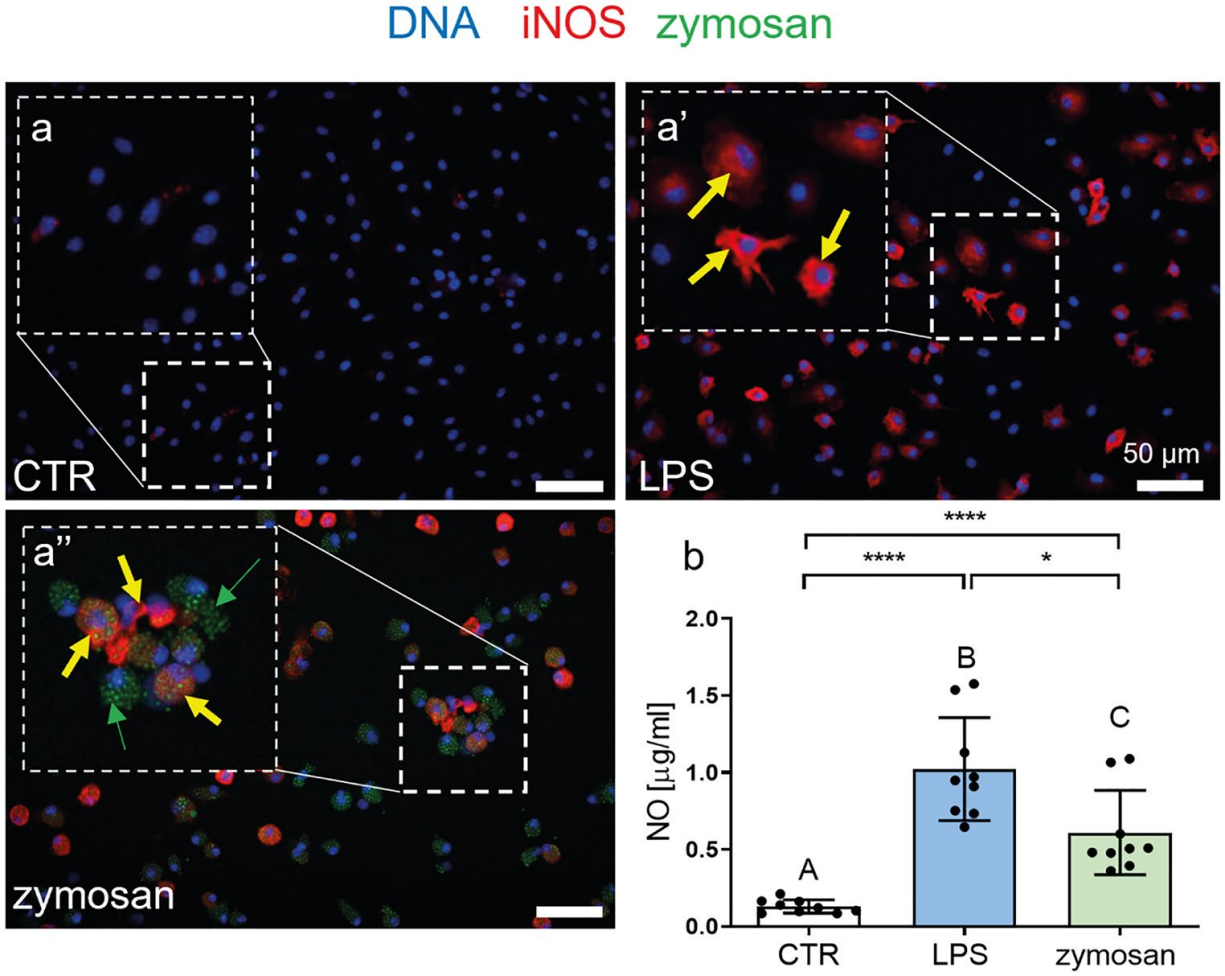
Inducible nitric oxide synthase (iNOS) expression and nitric oxide (NO) production by bone marrow-derived macrophages (BMDMs) following zymosan and lipopolysaccharide (LPS) stimulation. **a**–**a’’** Differentiated BMDMs were stimulated overnight with LPS (1 µg/ml) and zymosan (50 µg/ml) or left alone (CTR). Subsequently, Hoechst 33342 (blue) was used to stain their DNA, and iNOS expression was estimated by immunocytochemistry (red). **a’**–**a’’** Representative images showing iNOS expression by BMDMs after stimulation with LPS and zymosan. Exemplary iNOS^+^ positive cells are marked with yellow arrows, and zymosan particles (autofluorescent) are pointed with green arrows. Scale bar = 50 μm. **b** Quantification of NO production by BMDMs after stimulation with LPS and zymosan. NO production was determined with a Griess assay. Asterisks indicate significant differences using unpaired two-tailed Student’s *t* test (*P ≤ 0.05, ****P ≤ 0.0001) between experimental groups versus CTR and LPS versus zymosan. Different letters indicate statistically significant differences between groups using a one-way analysis of variance (ANOVA) (post hoc Tukey test). N = 3. Data are presented as the mean ± SD

Knowing that BMDMs indeed represent differentiated macrophages and upon stimulation are capable of MET release, we intended to verify some of the unexplored mechanisms of the trap formation and, in particular the involvement of endogenous nitrogen species. Nitric oxide is the principal oxide of nitrogen and its synthesis requires expression of iNOS (NOS2). For this reason, in the first step we verified iNOS expression (Fig. 4a–a’’); whereas untreated control BMDMs did not express it (Fig. 4a), either LPS or zymosan induced NOS2 (Fig. 4a’–a’’), although there was a tendency to a stronger response upon LPS (Fig. 4a’). This corresponded to the production of NO itself which was the strongest upon LPS stimulation and still enhanced by zymosan, yet significantly lower (Fig. 4b).

### MET formation occurs in a NO-dependent manner

**Fig. 5:**
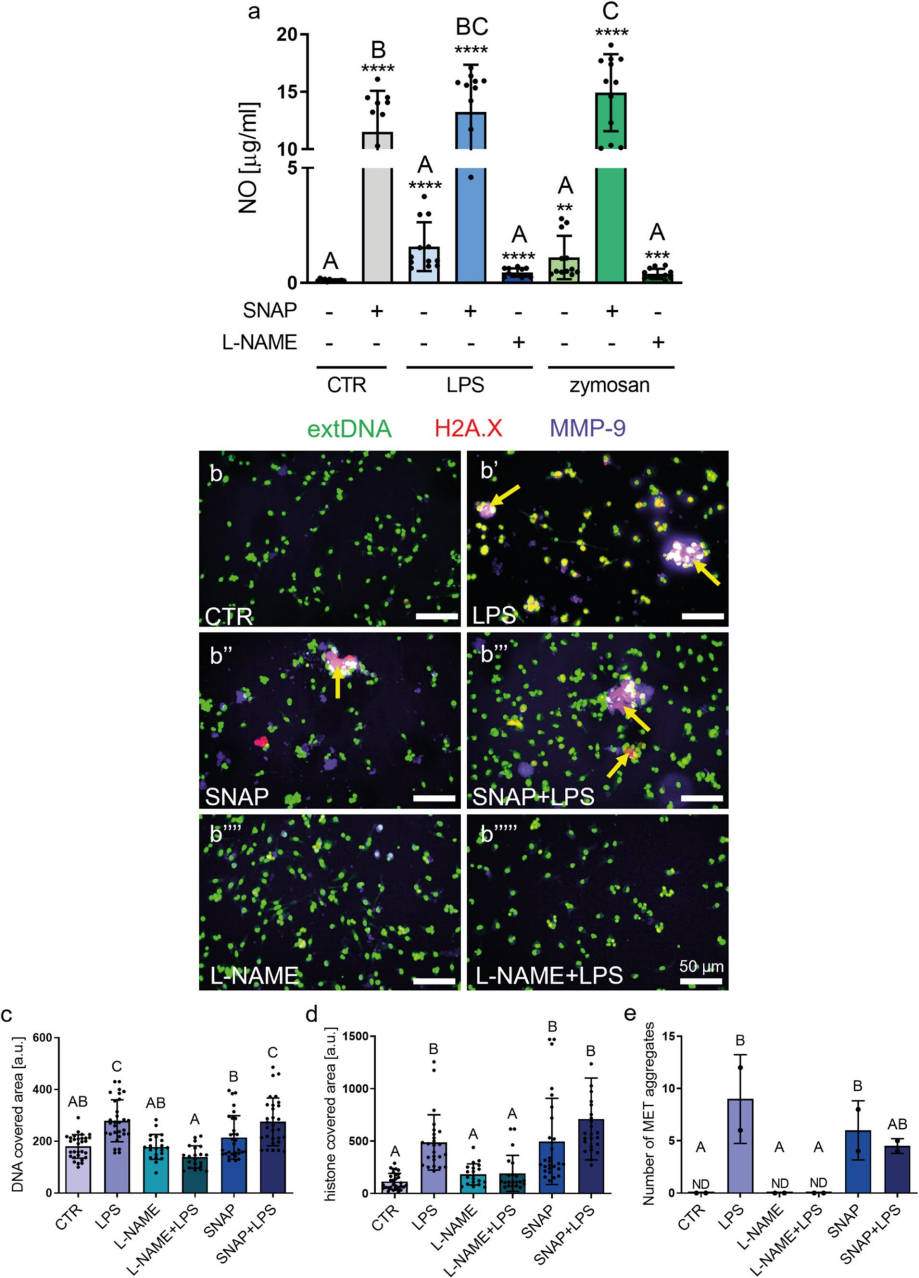
Donor (SNAP) and inhibitor (L-NAME) of nitric oxide (NO) impact macrophage extracellular trap (MET) formation by bone marrow-derived macrophages (BMDMs). Differentiated BMDMs were pretreated with SNAP (0.5 mM) or L-NAME (2 mM) for 30 min and then stimulated overnight with LPS (1 µg/ml), zymosan (50 µg/ml) or left alone (CTR) to induce NO production or MET formation. **a** Production of NO by BMDMs after stimulation with LPS or zymosan in the presence or absence of SNAP and L-NAME. NO production was determined with a Griess assay. Asterisks indicate significant differences using unpaired two-tailed Student’s *t* test (**P ≤ 0.01, ***P ≤ 0.001, ****P ≤ 0.0001) between experimental groups versus CTR without SNAP or L-NAME. Additionally, one-way analysis of variance (ANOVA) (post hoc Tukey test) was used to compare all groups (different letters indicate statistically significant differences between groups). Data are presented as the mean ± SD of triplicate experiments. **b**–**b’’’’’** Representative immunocytochemical images showing MET release and aggregates. BMDMs were pretreated with SNAP (0.5 mM) or L-NAME (2 mM) for 30 min and then stimulated overnight with LPS (1 µg/ml). Upon fixation, extDNA was stained with Sytox Green (extDNA; green), the expression of H2A.X histone (red) and MMP-9 (purple) by immunocytochemistry. The yellow arrows mark exemplary METs. Scale bar = 50 μm. **c**–**d** Quantification of MET components: **c** extracellular DNA and **d** histone covered area. **e** Quantification of the METs forming aggregates. **c**–**d** The area was quantified with ImageJ software and is expressed in arbitrary units (a.u.) per field of view. Different letters indicate statistically significant differences between groups using the one-way ANOVA (post hoc Tukey test). Student’s *t* test data overlapped with ANOVA analyses and it is not shown for clarity. N = 3. Data are presented as the mean ± SD. ND, not detected

In order to determine NO involvement in MET formation, first, we tested if BMDMs are sensitive to a NOS inhibitor (L-NAME), and if the cells respond to NO donor (SNAP), as we intended to use these tools in the following studies on METs. Indeed, SNAP alone generated large quantities of NO which was easily detectable in the medium, independently of the additional presence of LPS or zymosan, although in the latter case even higher NO levels were detected (Fig. 5a). On the other hand, when applied with LPS or zymosan, L-NAME showed a tendency to inhibit NO production by BMDMs, but this difference did not reach statistical significance (Fig. 5a). Thereafter, we used SNAP and L-NAME to verify the involvement of NO in MET formation by cryopreserved, thawed BMDMs. L-NAME alone did not induce MET release (Fig. 5b’’’’). However, when the cells were pre-treated with L-NAME and then stimulated with LPS, this dramatically decreased MET release: no extDNA or histone H2A.X was detected and (only a very weak MMP-9 signal was observed (Fig. 5b’’’’’, c, d; Supplementary Fig. 3)). Moreover, no MET aggregates were seen. In the studies presented in Fig. 5b–b’’’’’, we obtained images at lower magnification than in previous experiments, thus larger areas are visible. In such images, clusters or aggregates were seen as indicated by yellow arrows. Interestingly, when we applied exogenous NO (SNAP) into the system, it alone did induce MET release and formation of some aggregates (Fig. 5b’’, e; Supplementary Fig. 3) and when additionally LPS was added, the release of extDNA was even stronger (Fig. 5c) and there was a tendency to enhance presence of extracellular histones (Fig. 5d).

## Discussion

Several studies have shown that ET formation is a phenomenon commonly shared by innate immune cells to capture and eliminate pathogens, but ETs can also lead to pathological conditions (Nija et al. 2020). Thus far, majority of studies on ETs have been performed on traps released by neutrophils (NETs) and numerous mechanisms operating during their formation have been described (Tatsiy et al. 2021; Kenny et al. 2017; Kolaczkowska et al. 2015; Lewis et al. 2015). Although there has been progress in understanding mechanisms of ET formation, the existing knowledge is still incomplete, especially in the case of monocytes circulating in the blood, and this also applies to macrophages present in tissues and organs. The former cells are recruited to the sites of inflammation just after neutrophils, whereas resident macrophages engage their defense mechanisms even earlier, once the infection or injury has occurred (Silva 2010). Both neutrophils and monocytes/macrophages originate from common myeloid precursors in the bone marrow although subsequently their hematopoietic pathways diverge. Nevertheless, they share similarities in co-expression of some antigens, production of some granular proteins, oxidants, cytokines, chemokines, and both are vital for immunity by building and modulating innate responses (Geissmann et al. 2010). Therefore, in addition to the much more extensively studied and best-defined NETs, METs are also being investigated. In particular their potential contribution to the overall content/levels of extracellular DNA (e.g. MPO-DNA complexes detectable in plasma) in some pathological states (Granger et al. 2017; Hanata et al. 2022) is of interest. However, the similarities between the two types of ETs make the studies challenging. The type of macrophages used in such studies also needs to be considered. This is because primary macrophages isolated directly from the organism as well as macrophage cell lines have predetermined functions or polarization status (Murray and Wynn 2011) which might pose an obstacle in establishing a satisfactory model relevant for MET studies. Macrophage polarization has been important in some MET studies performed on HMDMs revealing M1 being more MET-prone than M2 (Rayner et al. 2018; Zhang et al. 2019). However, in our studies, we wanted to investigate the mechanism of MET formation by BMDMs that originated from naïve BM cells, so we did not prime/polarize them into M1 or M2 phenotypes. Hence, we aimed at establishing a suitable model based on naïve macrophages such as BMDMs differentiated *ex vivo* from BM cells. Moreover, since the goal was to make this model as practical as possible avoiding the need for freshly isolated mouse BM cells for differentiation (each time a mouse would have to be sacrificed), we utilized the method of freezing BM cells and differentiating BMDMs from thawed BM cells. Indeed, by this approach, we successfully obtained differentiated BMDMs that had a typical macrophage morphology and expressed the F4/80 marker as confirmed by flow cytometric analyses and by immunocytochemistry. According to the flow cytometry results, there was a tendency to an increased F4/80 expression (shift from app. 11 to 40% positive cells); however, it did not reach statistical significance. Thus, to confirm BMDM differentiation, we also performed analyses of CD11a/CD18 (LFA-1) antigen expression alone and as a co-expression with F4/80. CD11a is one of four β2 integrins expressed on macrophages (Schittenhelm et al. 2017). We found out that the majority of BMDMs expressed LFA-1 and almost all F4/80^+^ cells were also CD11a/CD18^+^. Although at this point, we do not know why flow cytometric analyses indicated lower expression of F4/80 than immunocytochemical one, the surface presence of LFA-1 confirmed the expected differentiation and is in line with literature on BMDMs (Vereyken et al. 2011). On the other hand, immunocytochemistry showed that the majority of nucleated cells (BMDMs) expressed that marker. We do not know what is the reason for this discrepancy but our further analyses confirmed not only the morphological differentiation of these cells into macrophages but most importantly, the functional one. In line with this, BMDMs were actively phagocytizing zymosan particles, expressing iNOS in response to various stimuli and releasing NO.

Knowing that BMDM cells reached the state of mature macrophages, we tested their capacity to cast METs upon three different immunostimulants: LPS, zymosan and PMA. LPS is the major component of Gram-negative bacterial cell wall and is known to be a strong activator of macrophages in terms of interaction with Toll-like receptors 4 (TLR4) expressed on these cells and responsible for their transformation into a pro-inflammatory phenotype (Zanoni et al. 2011; Haim et al. 2014), whereas yeast zymosan is an extract from *S. cerevisiae* cell wall containing various polysaccharides, including several ß-glucans and is recognized by TLR2 also present on macrophages (Underhill 2003). Lastly, PMA — diacylglycerol (DAG) mimetic, is a chemical agent activating protein kinase C (PKC) which alters expression of the nuclear factor-κB (NFκB) leading to expression of pro-inflammatory cytokines (Gray et al. 2013; Desai et al. 2016). All 3 stimuli have been previously shown to induce NET release (Neeli et al. 2009; Brinkmann and Zychlinsky 2012; Schauer et al. 2014) as well as METs by several types of macrophages (Doster et al. 2018a). To test the capacity of our BMDMs differentiated from frozen BM cells to release METs, at first, we examined their formation prior to fixation required by immunocytochemistry. This method of detecting METs allowed us to see what the traps look like without changing their morphology which might occur upon fixation. Indeed, after stimulation with LPS, zymosan or PMA, we observed METs and they appeared as elongated and intertwined extDNA strands, but in some cells, we could also see enlarged nuclei without attached extDNA strands. We postulate that the latter represent the first step of MET formation process and should be interpreted as preparation of some cells to release them. This 2-step process was originally shown for NETs when Rodríguez-Espinosa et al. (2015) reported that in the absence of glucose (or upon glycolysis inhibition) PMA-stimulated neutrophils are unable to release NETs, but their nuclei display altered morphology with a loss of nuclear integrity and chromatin decondensation. When glucose was added back in, these cells released NETs completing the whole process (Rodríguez-Espinosa et al. 2015). Therefore, it might be that if we increased incubation time, macrophages with decondensed chromatin would eventually cast METs when glucose and other nutrients were present in the culture media.

Interestingly, when traps are cast extracellularly, their morphology might vary. In the case of PMA-induced NETs, their structure can be diffused and spread (Gray et al. 2013) but some describe them as cloud-like NETs (Brinkmann and Zychlinsky 2012). When it comes to LPS stimulation, similar terms are used “cloudy NETs” or “cloudy NETs with spikes” (Maueröder et al. 2016; Sosa-Luis et al. 2021). Additionally, under high neutrophil densities, NETs aggregate and start performing anti-inflammatory roles reflected by cytokine/chemokine degradation via proteases present in their structure (Schauer et al. 2014). In our studies, we observed that upon stimulation with LPS, zymosan or PMA macrophage extracellular traps did differ in their morphological structure and appearing as either elongated extDNA strands or diffused extDNA (similar to “cloudy NETs”). We also noticed that METs sometimes formed aggregates and we observed it mostly in immunocytochemical studies preceded by fixation. Nevertheless, MET aggregates were not formed in large numbers, but rather occasionally, although the density of cells was always the same. Therefore, we hypothesize that such aggregates can be formed naturally, however, fixation might also cause their clustering being an artefact.

In terms of response of macrophages to various stimuli resulting in MET formation, our results differ from some other studies. For example, Liu et al. (2014) reported that mouse peritoneal macrophages did not produce METs upon stimulation with LPS or PMA (Liu et al. 2014). These contradictory results may be due to differences between BMDMs (differentiated *ex vivo*) and peritoneal macrophages (differentiated and recruited *in vivo*), as the latter are already pro-inflammatory primed with thioglycolate which is injected into mice to recruit them to the peritoneal cavity (Turchyn et al. 2007; Liu et al. 2014; Pavlou et al. 2017). Such cells might represent an exhausted phenotype of macrophages unable to respond to the second stimulation. Indeed, Zajd et al. (2020) compared the two cell types head-to-head and found out that BMDMs are more phagocytic, have significantly upregulated expression of surface receptors upon stimulation, and produce more cytokines and chemokines in comparison to peritoneal macrophages (Zajd et al. 2020). Also of note is that we isolated cells from C57Bl/6 J mice with dominant cellular response, whereas Liu et al. (2014) collected their peritoneal macrophages from BALB/c mice with dominant humoral immunity (Kolaczkowska et al. 2001; Sahputra et al. 2022). Moreover, we used male mice whereas Liu et al. (2014) female mice, and it has been shown that the former macrophages are more susceptible to inflammatory stimuli (Barcena et al. 2021). In fact, in our studies, we detected stronger MET formation upon LPS than other stimulants and in particular zymosan; however, we are unaware of other studies on any type of macrophages that would simultaneously compare effects of the three stimulants used herein. Diverse responses to different stimuli are expected as in the case of NETs, distinct responses can be triggered by various stimuli (Kenny et al. 2017).

An interesting observation was also made in regard to METs and phagocytosis of zymosan particles by BMDMs. Thus far, it was speculated that in the case of neutrophils, they either phagocytize or cast NETs, rather than do it simultaneously (Branzk et al. 2014; Castanheira and Kubes 2019). It also explains why only some neutrophils make NETs during infection and why neutrophils that phagocytose bacteria do not subsequently release NETs as bacteria could escape them during lytic NET formation. Therefore, our observation that macrophages can perform both those processes simultaneously — as we did observe after zymosan stimulation (the largest and autofluorescent stimulant used in the study) — might represent a distinctive feature of MET, which release could more likely accompany phagocytosis. Previous studies on peritoneal macrophages revealed that the capacity to perform phagocytosis by primary non-elicited (resident) macrophages was less effective, yet ongoing, than that of elicited macrophages (Pavlou et al. 2017). Considering the naïve status of our BMDMs, we could expect some phagocytosis to occur and indeed this was confirmed in the studies with zymosan. Moreover, it has been shown that NO donors, inducing SNAP, have no impact on phagocytosis by human neutrophils although they augment ROS production and bacterial killing (Kumar et al. 2010). In our studies, we observed that zymosan-stimulated BMDMs actively phagocytosed zymosan particles and produced NO suggesting that NO and RNS did not alter the phagocytic activity of BMDMs.

Once we confirmed that BMDMs were releasing extDNA indicative of METs and assessed their morphology, we introduced another technique to confirm that extDNA was decorated with proteins validating that they were indeed METs. This is because extDNA alone can be an artifact (due to the cell handling) or indicative of cell death resulting in necrotic morphology (Boeltz et al. 2019). In line with this, we confirmed by immunocytochemistry that METs formed by BMDMs contained nuclear and granular proteins and in particular histone H2A.X and MMP-9. The two proteins co-localized within extDNA constituting the MET scaffold which was further confirmed by recreating a 3D METs model revealing that these proteins not only co-localized but also were entwined and connected with extDNA.

Our next goal was to unveil some of the mechanisms operating in MET formation and in particular the involvement of RNS in it as very little is known in this respect. Thus far, involvement of ROS was identified only in the case of human, mouse and bovine macrophages, THP-1 and RAW 264.7 cells. In regard to the former, ROS formation was first confirmed in human alveolar macrophages stimulated with nontypeable bacteria *Haemophilus influenzae* (NTHi) and then the authors showed ROS involvement in MET formation by application of NADPH oxidase inhibitor — apocynin (King et al. 2015). This was also confirmed for bovine macrophages stimulated with PMA or bacteria *Mannheimia haemolytica* via inhibition of NADPH oxidase with diphenyleneiodonium chloride (DPI) resulting in decreased MET formation (Aulik et al. 2012). DPI also reduced MET formation after stimulation with heme-activated platelets (Okubo et al. 2018), aflatoxin B1 (An et al. 2017), *Staphylococcus aureus* (Shen et al. 2016), and *Streptococcus agalactiae* (Doster et al. 2018b), indicating ROS-dependent MET release. Contrary to these studies, ROS-independent MET induction was reported in the case of *Candida albicans* (Loureiro et al. 2019), *Escherichia coli* (Liu et al. 2014), and *Mycobacterium tuberculosis* (Kalsum et al. 2017; Wong and Jacobs 2013) challenge, and DPI did not decrease MET formation. However, to date, no studies have verified the role of RNS in MET formation. We have preselected them as potentially involved in this process as their contribution has now been confirmed in the case of NETs. For example, Manda-Handzlik et al. (2020) demonstrated that human neutrophils isolated from peripheral blood and stimulated with either by a NO donor — SNAP or a NO metabolite — peroxynitrite, formed NETs. Moreover, NET formation was enhanced upon subsequent stimulation with platelet activating factor (PAF), LPS, calcium ionophore (CI) or PMA (Manda-Handzlik et al. 2020). To prove RNS involvement in MET release, the nitrogen species were then inhibited with L-NAME or RNS scavengers (Manda-Handzlik et al. 2020). Being aware of the above studies, we applied selected tools tested in the above studies to verify whether RNS are also involved in MET formation. Firstly, we confirmed that BMDMs expressed iNOS and produced NO upon stimulation with LPS and zymosan. NO production in the presence of pro-inflammatory stimuli depends on iNOS activity whose expression is up-regulated in the inflammatory environment (Kröncke et al. 2001). Inducible NOS converts L-arginine to L-citrulline and free radical NO (Alderton et al. 2001). Nitric oxide is short-lived, thus its relatively stable end products are being detected, and it is NO_2_^−^ in the Griess method whose sensitivity is in the micromolar range (Möller et al. 2019). Of the available tools, we selected a NOS inhibitor L-NAME, and a NO donor SNAP. L-NAME is a non-selective NOS inhibitor which is hydrolyzed by cellular esterases to Nω-Nitro-L-arginine (L-NNA) to become fully functional and efficient in the inhibition of NOS (Griffith and Kilbourn 1996). L-NAME is widely used in *in vitro* experiments investigating the result of limitation of NO production (Kopincová et al. 2012). Our results demonstrated that inhibition of endogenous NO production by L-NAME not only decreased the NO content itself but most importantly impaired MET formation by LPS stimulated BMDMs — both at the backbone and protein levels, respectively, as much less extDNA and histone H2A.X were released upon pretreatment with L-NAME. Also, no MET aggregates were observed any more. To contrast that, in some studies, we stimulated cells with a NO donor itself or in combination with stimulants to see the impact of exogenous NO on BMDMs. SNAP is an S-nitrosothiol which in aqueous solutions/physiological environment undergoes spontaneous hydrolysis to disulfide and NO, which is slowly released (Chipinda and Simoyi 2006). We knew from the literature that exogenous NO diffuses rapidly through cell membranes due to its lipophilicity (Denicola et al. 1996), however, other studies showed that SNAP can also enter into lipid membranes, and its location is limited to the hydrophobic core of membrane bilayers, which allows NO to diffuse freely across membranes, showing its effectiveness *in vitro* and *in vivo* (Nedeianu et al. 2004). In line with this, we detected that SNAP alone induced MET formation (extDNA + histone H2A.X) and also MET aggregates. The pattern and intensity of these traps formation were similar to LPS-induced structures and when the two were added together to macrophages, the extDNA release was even stronger than upon SNAP alone.

Overall, our study shows that BMDMs obtained from frozen bone marrow cells represent a good and relevant model to study naïve, unpolarized macrophages in terms of MET formation and its mechanisms. Most importantly, we show that both endogenous and exogenous RNS are important inducers of MET formation and thus that macrophage extracellular traps are indeed formed in an RNS-dependent manner.

## Conclusion

In summary, the study demonstrated that BMDMs obtained from cryopreserved, thawed bone marrow cells represent morphologically differentiated and functional macrophages which are able to form METs upon stimulation with various immunostimulants. Above all, the study showed for the first time, involvement of reactive nitrogen species and in particular of nitric oxide in MET formation. Although detailed mechanism(s) remain to be elucidated, the study paved the way for further investigations. It also confirmed that one more mechanism of the extracellular trap formation is shared by granulocyte and monocyte/macrophage lineages.

### Supplementary Information

Below is the link to the electronic supplementary material.

Supplementary file1 (PDF 511 KB)

Supplementary file2 (MP4 13324 KB)

## Data Availability

All datasets used and/or analyzed during the current study are available from the corresponding author on reasonable request.
