# Supplementary material for: Impact of endogenous and exogenous nitrogen species on macrophage extracellular trap (MET) formation by bone marrow–derived macrophages
Source: Cell Tissue Res. 2023 Oct 4;394(2):361–77. doi: 10.1007/s00441-023-03832-z (PMC10638184; doi:10.1007/s00441-023-03832-z)
Supplement: Supplementary file 1 — Supplementary file1 (PDF 511 KB) [file 441_2023_3832_MOESM1_ESM.pdf]

A

B

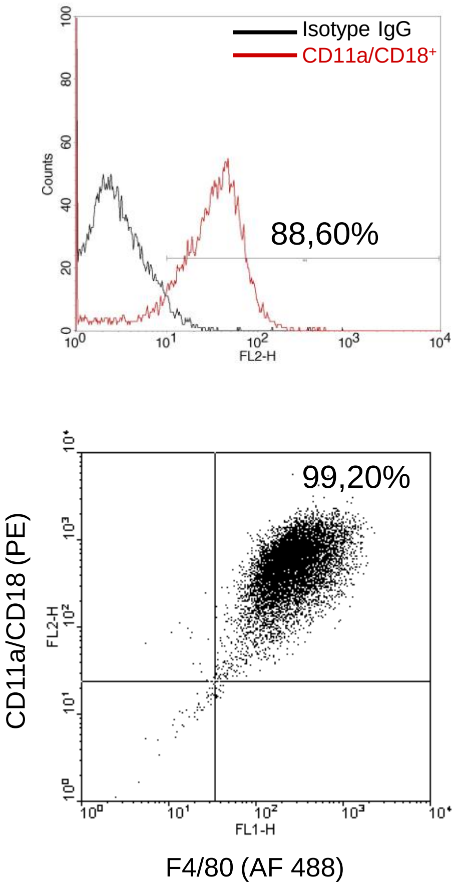

Supplementary Figure 1

Representative data confirming expression of CD11a/CD18 on thawed BMDM cells. (A) Majority of the cells were expressing CD11a/CD18 (antibodies conjugated with PE, channel 2 (FL2-H)); the black line represents the signal of the isotype control antibody, and the red line marks the fluorescence intensity of the CD11a/CD18$^+$ cells. (B) Co-expression of F4/80 (antibodies conjugated with Alexa Fluor 488, channel 1 (FL1-H)) and CD11a/CD18 antibodies in FL2-H.

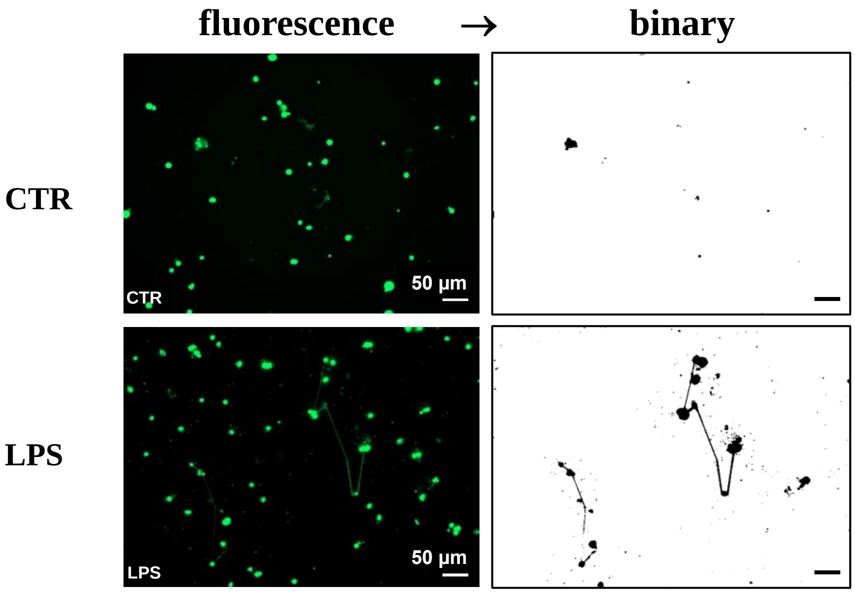

Supplementary Figure 2

Quantification of METs in ImageJ software. Fluorescent Sytox Green signal from METs was estimated by ImageJ software with the presented approach. In the ImageJ, thresholded images with adjusted brightness/contrast were changed into binary (black and white) images and then the nuclear signal only (condensed nuclei) was removed prior to analysis; next the software measured only extracellular DNA located outside of the cells. On the left hand side, exemplary images prior processing and on the right hand side, after processing. CTR – untreated control cells; LPS – cells stimulated with lipopolysaccharide. Scale bar = 50 µm.

Supplementary Fig. 3

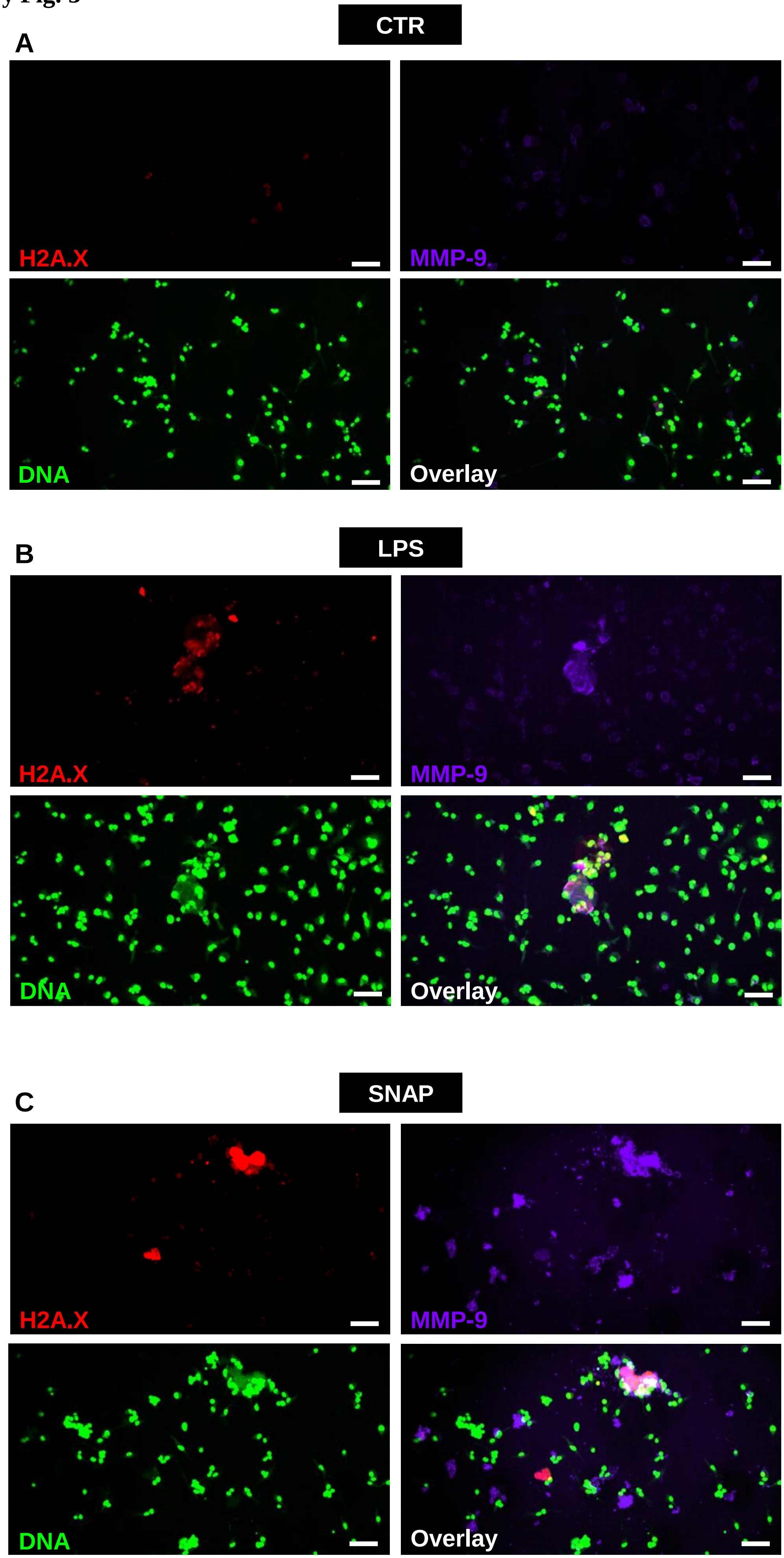

Supplementary Fig. 3 – cont.

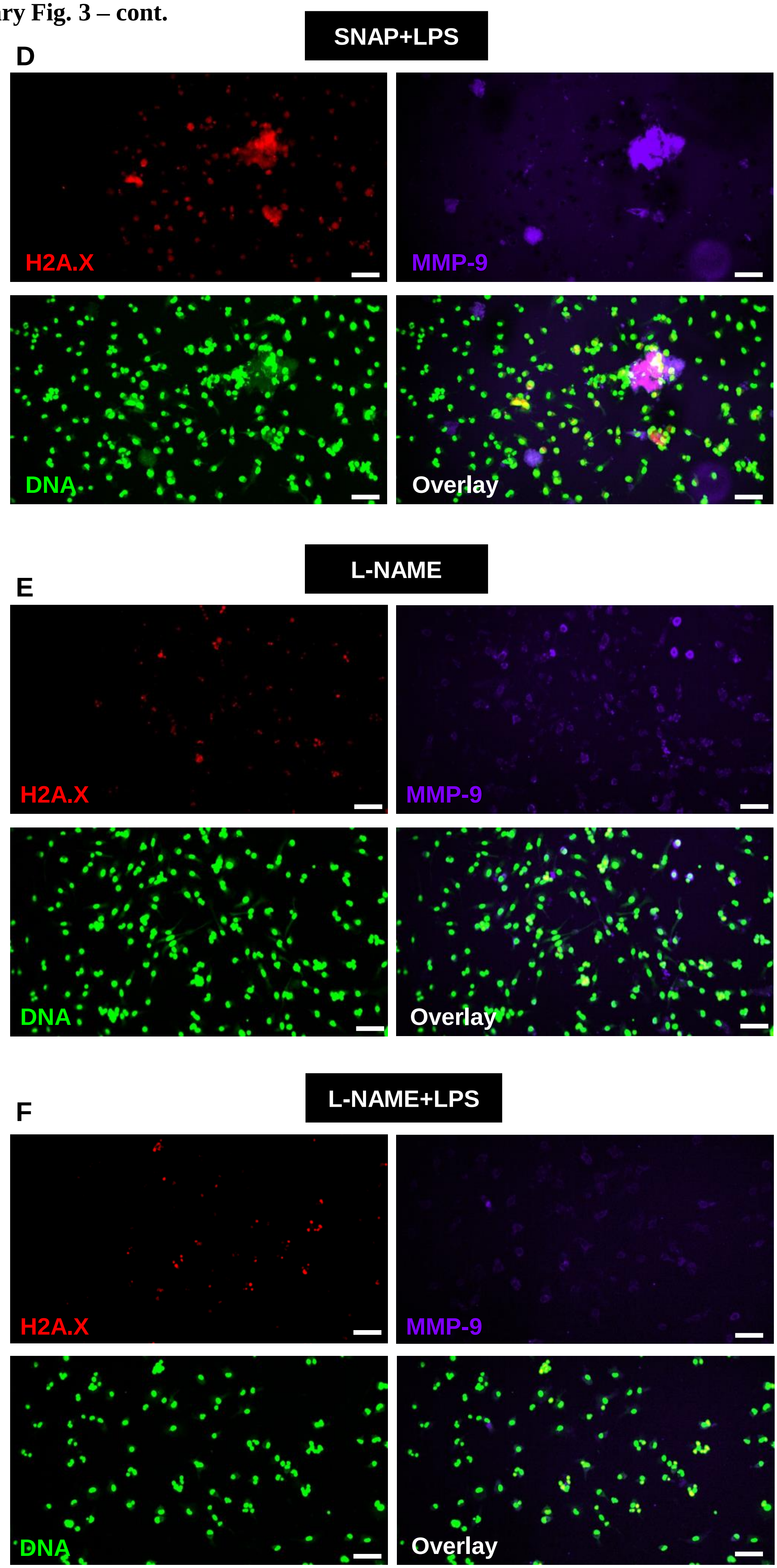

Supplementary Figure 3

Donor (SNAP) and inhibitor (L-NAME) of nitric oxide (NO) impact macrophage extracellular trap (MET) formation by bone marrow-derived macrophages (BMDMs). (A-F) Representative immunocytochemical images, from single chanels and overlayed, showing MET release and aggregates are shown. BMDMs were pretreated with SNAP (0.5 mM) or L-NAME (2 mM) for 30 minutes and then stimulated overnight with LPS (1 μg/ml). Upon fixation, extDNA was stained with Sytox Green (extDNA; green), and then the expression of H2A.X histone (red) and MMP-9 (purple) by immunocytochemistry. Scale bar = 50 μm.
